# Statistical Reports of the Health of the Navy, for the Years 1830—36 Inclusive East India Command

**Published:** 1842-07-01

**Authors:** 


### Statistical Reports of the Health of the Navy, for the Years 1830—6 inclusive.

### East India Command.

In our last number we gave an account of the Cape and African Command. We now proceed to that of the East Indies. This is a command of vast extent, embracing upwards of 70 degrees of latitude, and 100 of longitude—extending from the tropic of Cancer to New Holland, &c. and bounded on the North by the Isthmus of Suez. Still the operation of our ships is chiefly directed to the shores of the bay of Bengal—the coast of Malabar—and the Island of Ceylon. The Eastern Isles, and China, *now* occupy many of our ships. The station may be considered as, in a large proportion, intertropical.

Affections of the alimentary canal, including cholera, are the most frequent, and also the most fatal. Next to these are hepatic diseases—" in many instances closely connected with the former." These hepatic affections, though they do not occasion so much mortality on the station, yet induce a great deal of invaliding, and thus keep up a constant drain on the force. They are designated as " inflammatory, acute, or chronic," in the nosological returns; though the terms are often improperly employed. Idiopathic fever, of an endemic character, does hold a very prominent rank in the catalogue.

" It is an interesting question, to the solution of which little, in effect, has yet been contributed, why one disease, or class of disease, prevails in one place, and another, in another. Why, for example, is dysentery, not fever, the most fatal disease of the East? Why, on the West coast of Africa, and at Jamaica, is there little mortality from any disease but fever? And, why is the fever different in the two places? Why, on the other hand, is there no epidemic, and little endemic disease on the coast of Brazil? the maladies which occur there being the effect of simple atmospheric action, errors in diet, exercise, and the abuse, or improper application of what the ancients quaintly called ' non-naturals.' It must be clear, to the most superficial observation, that the attempts, where attempts have been made, to explain these, and similar results, have entirely failed." 78.

### 1830.

The mean force of the year 1830 was 1,621, dispersed in 14 vessels—4 frigates and 10 smaller vessels. The total number of sick and hurt was 2,520—at the high rate of 1,548 to the 1,000. The succeeding ratios, however, indicating the *results* of diseases and accidents, are not high, when intertropical service is considered. 86 cases were sent to hospitals—25 were invalided—and 29 died, 19 on board ship, and 10 in hospitals—or about 18 in the thousand. The total loss to the squadron was about 33 per 1,000—little more than one-third of the loss on the Cape and African station, in the same year.

Under the head of " Idiopathic fevers," 174 cases were treated, of which number, nine were sent to hospital, and three were invalided for the sequelæ of fever. In a great majority of cases the fever was very slight and transitory. They were divided into 108 of continued—18 of remittent—and 48 of intermittent fever. Six of the first class were sent to hospital, and one invalided. Of the second class (remittents) 3 were sent to hospital—and of the third class (intermittents) 1 was invalided. No case terminated fatally at sea, and 1 only in hospital. Thus 162 cases were cured at sea—and 8 in hospital—the total loss from fever in the squadron being 4—3 invalided, and 1 death.

In the Crocodile, soon after anchoring in the roads of Batavia, on her way from Australia to India, 17 cases of remittent fever broke out *simultaneously!* It was in the month of August—the days excessively hot—the nights damp and chilly. None died; but it is evident that emanations from the shore were the chief agents in this outbreak, aided, no doubt, by atmospheric vicissitudes.

"Inflammation with fever" was an order of diseases, more numerous and important; because, in the nosological returns, it included hepatic affections. The numerical amount is swelled by including all inflammations of external parts, however trifling. There were 707 cases this year in the squadron, of which 28 were sent to hospital—14 were invalided—and seven died. There were 49 cases of inflammation of the lungs, 3 went to hospital, and one died. There were 78 cases of inflammation of the liver. 11 were sent to hospital—6 were invalided—and 5 died, 4 on board and 1 in hospital. Hepatic disease, though so prevalent in India as to be called the "endemic" of the country, is yet not so fatal as disease of the alimentary canal. No case of phthisis was noted in the year. Of 184 cases of dysentery, 11 died. Three out of 5 cases of cholera proved fatal.

There were 26 cases of scurvy—now a rare disease in the navy. They occurred in two vessels employed on the west coast of Australia. For a considerable time the ships' companies had had little fresh meat or vegetables. *They had lemon-juice in abundance.* When fresh meat and vegetables were procured, the disease vanished.

1831.

The ratio of mortality this year was low—about 13 in the thousand men—though the ratio of sickness was very high. The ratio of invaliding was enormous—viz. about 60 in the thousand. The mean numerical force this year was 1,523—the number of sick and hurt 2,549—or 1,673 for every thousand. There were 20 cases of cholera, 6 of which were fatal on board. Two were sent to hospital. Four of the fatal cases were in the Cruizer, on her passage from India to Swan River. They had all the same characters, but happened at long intervals, and under different circumstances. The first case happened at Bombay, in May. The second on the 12th of July—the third and fourth on the 26th of July—and the fifth on the 8th of August. This was a curious specimen of a contagious disease.

Passing over 1832, we shall dwell for a moment on

1833.

In the UNDAUNTED, frigate, there were 98 cases of cholera, and eight deaths. The ship had come from the Cape of Good Hope, in high health, and had been 14 days in Madras roads when the cholera burst forth—ran its course—and terminated in 11 days. "The surgeon gives a negative opinion as to its possessing contagious qualities." The remaining years present nothing particular, except a progressive decrease of mortality.

In the whole seven years there were only 39 deaths form FEVER—or, about three in the thousand per annum of men employed. On the South American Station, the rate of mortality from FEVER, however, was only one in the thousand, per annum, of force on service!!

Let us now glance at the "HOME and VARIOUS" Station. The home force is divided into two classes—vessels that remain permanently on the home station—and those which are sent with despatches abroad, and return almost immediately. The vital statistics here, are somewhat complicated. The remarkably salutary influence of "change of air," or of climate, will appear from the following passage.

"During the year 1830, the ships on the 'Home' station were highly healthy; those employed 'Variously' were, however, greatly more so; it may rather be said that they enjoyed a singular immunity from fatal disease. There is something surprising in the amount of difference, especially when the nature of the various employments is considered." 149.

"The most important result, viz. of proportionate mortality in each, is very remarkable. As has been stated, it is low in both, singularly so in the force employed 'Variously.' In the ships constantly on Home stations, it is, from all causes, in the ratio of 8·7 per 1,000 of strength; from disease, independent of external causes, 6 per 1,000. But, in ships employed 'Variously,' the rate of dying from all causes, is 6·4, and from disease no more than 3·4 per 1,000 of strength; being little more than half that sustained by the 'Home' class, and so low, however, and by whatever law tried, as to render it almost doubtful of belief, could there be any doubt, as to the means by which it has been determined." 150.

Thus the ratio of mortality in ships employed in harbours and about the coasts at home, is nearly double that which occurs in ships employed occasionally in traversing the ocean with despatches.*

The ships on the Home Station, during the seven years alluded to, suffered less from idiopathic fever than any other division of the naval force. The average number attacked was 51 in 1,000—the average of deaths was 1 in 1,188 of the force annually. In the "VARIOUS" force, the ratio of attacks was higher, viz. 60 in the 1,000, and the average of deaths was nearly double that of the HOME Service. The latter is a little higher than on the South American Station—a little lower than in the Mediterranean—and about half that of the East Indies.

But the following table will shew that the ratio of sickness, as well as of mortalilty, was less in the East Indies, considerably, than at home!

| | Attacked per 1,000 of Force. | Died per 1,000 of Force. |
|---|---|---|
| East Indies | 2·9 | 1·2 |
| Home | 4·1 | 1·4 |

* This applied to the year 1830; but on the whole aggregate of the seven years, the "Various" did not maintain this superiority. The ratio of sickness in both classes was about the same.

| | Attacked per 1,000 of Force. | Died per 1,000 of Force. |
|---|---|---|
| South America | 3·2 | 1·5 |
| Africa | 3·4 | 1·5 |
| Various | 4·2 | 1·8 |
| West Indies and North America | 4·8 | 1·9 |
| Mediterranean | 5·1 | 1·9 |

"From this view, it appears that the mortality was least in the East Indian, greatest in the West Indian and North American, and Mediterranean, force; but the loss in the greatest, being under 2 per 1,000 annually of the employed, is not heavy, when compared with that sustained by many other portions of society at corresponding ages. Although the proportion of mortality in the West Indian and North American, and Mediterranean, commands, was the same, the proportion of attacks in the latter preponderated: it therefore appears, that the latter division of cruizing ground is the least favourable of any to consumptive disease; for, besides the greater frequency, there was also a larger proportion of invaliding. As a part of the invaliding was from 'Home' hospitals, it may be assumed that some of the subjects so disposed of eventually died, without any means of tracing them. The East Indian was evidently the most favourable, the proportion of invalided, as well as of attacked and dead, being lowest. While that station is shown to be so favourable in respect of consumption, inflammation of the lungs, and other diseases of the respiratory organs, it is much the most unfavourable in those of alimentation." 208.

We must now dismiss this important volume, being unable to notice the numerous and valuable tables with which it is filled. We return our best thanks to the compiler of these Statistical Reports, as well as to the Physician-General of the Navy, under whose auspices and inspection they have been constructed.

